# Concurrence of clozapine-induced diabetic ketoacidosis and neuroleptic malignant syndrome: A case report

**DOI:** 10.1097/MD.0000000000044172

**Published:** 2025-08-22

**Authors:** Min Hyeok Shin, Jin Woo Jeong, Tae Yang Yu

**Affiliations:** a Department of Medicine, Division of Endocrinology and Metabolism, Wonkwang University School of Medicine, Iksan, Republic of Korea.

**Keywords:** clozapine, diabetic ketoacidosis, neuroleptic malignant syndrome

## Abstract

**Rationale:**

Clozapine is a unique antipsychotic drug used to treat treatment-resistant psychosis. Clozapine can induce metabolic complications and weight gain, and may lead to acute diabetic complications such as diabetic ketoacidosis (DKA). Neurological side effects of clozapine are relatively rare compared with those of typical antipsychotics; however, several cases have been reported. In particular, reports have suggested that clozapine-induced neuroleptic malignant syndrome (NMS) may present with atypical features. While multiple reports document DKA and NMS occurring separately in patients receiving clozapine, no cases have been reported in which they occur simultaneously. We report a case in which DKA and NMS developed concurrently, with NMS manifesting atypical features and the significant stress associated with NMS hindering recovery from DKA.

**Patient concerns:**

A 23-year-old man presented to the emergency department with generalized weakness and shortness of breath. He had been diagnosed with schizophrenia 3 years earlier and was taking medications prescribed at another hospital. His regimen was changed 2 months prior to presentation.

**Diagnosis:**

Arterial blood gas analysis revealed high anion gap metabolic acidosis and elevated blood ketone levels. The patient was diagnosed with DKA, and intensive insulin therapy was initiated. However, he developed fever and altered mental status during treatment, and despite intensive insulin therapy, the improvement of metabolic acidosis was hindered. Although the typical symptoms of NMS, such as rigidity and a rapid increase in creatine kinase (CK), were not observed, based on the history of clozapine treatment, persistent fever, altered mental status, and blood pressure instability, NMS was diagnosed.

**Interventions:**

Insulin and intravenous fluid therapy were continued for DKA, and clozapine was discontinued.

**Outcomes:**

Following clozapine discontinuation, metabolic acidosis, fever, and altered mental status improved rapidly.

**Lessons:**

DKA and NMS share various signs, such as fever, altered mental status, blood pressure instability, dyspnea, and tachycardia. Therefore, in patients receiving clozapine, the simultaneous occurrence of DKA and NMS should be considered. Additionally, clozapine-induced NMS may present in an atypical form; therefore, even in the absence of typical signs, such as rigidity and a rapid increase in CK, the possibility of NMS should be reconsidered.

## 1. Introduction

Clozapine, an atypical second-generation antipsychotic, is the most effective treatment for schizophrenia that is resistant to other antipsychotics.^[1]^ However, in addition to agranulocytosis, clozapine is associated with several life-threatening adverse effects.^[2,3]^ It can increase the risk of metabolic syndromes, including obesity, hyperglycemia, and dyslipidemia,^[4]^ which can lead to severe complications such as diabetic ketoacidosis (DKA).^[5]^ In particular, clozapine carries a higher risk of metabolic syndrome than other second-generation antipsychotics.^[6]^

Although clozapine has a lower incidence of neurological adverse effects than first-generation antipsychotics, neuroleptic malignant syndrome (NMS) can still occur.^[7]^ Clozapine-induced NMS may present atypically, distinguishing it from NMS caused by other antipsychotics.^[8,9]^

DKA and NMS share several features, such as dehydration, altered mental status, and tachycardia, which can complicate differential diagnosis.^[10,11]^ Rarely, they can even occur simultaneously.^[12]^ In such cases, the significant stress associated with NMS can hinder recovery from DKA. However, to date, no cases of concurrent DKA and NMS due to clozapine have been reported.

This case report describes a patient who developed simultaneous DKA and atypical NMS while receiving clozapine therapy, accompanied by delayed recovery from ketoacidosis.

## 2. Case report

A 23-year-old man presented to the emergency department with generalized weakness and shortness of breath. He had been diagnosed with schizophrenia 3 years earlier and was taking medications prescribed at another hospital. The regimen was modified 2 months prior to presentation. Over the past month, he had experienced significant weight loss and reported excessive thirst, frequent urination, and worsening shortness of breath the day before his visit.

His previous medication for schizophrenia included alprazolam (0.75 mg), ropinirole (2 mg), benztropine (1 mg), quetiapine (300 mg), amisulpride (800 mg), sertraline (100 mg), triazolam (0.25 mg), and etizolam (1 mg). Two months prior to visit, his treatment was revised to clozapine (100 mg), ropinirole (2 mg), benztropine (1 mg), quetiapine (100 mg), and amisulpride (400 mg). He had no history of other medication use, hypertension, diabetes, or significant medical or surgical conditions. He was a nonsmoker and did not consume alcohol. Over the past year, his weight had steadily increased to 105 kg, but he experienced rapid weight loss in the past month, measuring 88 kg the day before presentation. The patient had no family history of psychiatric conditions or metabolic syndrome, including diabetes.

On physical examination, the patient appeared acutely ill and his mental status was alert. His initial vital signs were as follows: blood pressure 148/79 mm Hg, pulse rate 138 beats/min, respiratory rate 22 breaths/min, temperature 37.7°C, height 170 cm, weight 87 kg, and body mass index 30.1 kg/m^2^. Table 1 shows the initial laboratory findings. These findings were consistent with those of severe metabolic acidosis due to DKA with rhabdomyolysis. Electrocardiography revealed sinus tachycardia at a rate of 140 beats/minute. Imaging studies, including chest radiography, abdominal radiography, chest computed tomography (CT), and abdominal CT, revealed no significant abnormalities.

**Table 1:** Summary of the initial laboratory test result.

Laboratory findings	Value	Unit	Reference range
Hemoglobin	15.4	g/dL	13.0–16.4
Hematocrit	42.9	%	39–49
White blood cell	12.06	10^3^/μL	4–10
Neutrophil (ANC)	10.47	10^3^/μL	1.5–7.5
Platelet	213	10^3^/μL	140–400
Glucose (serum)	500	mg/dL	70–100
Osmolarity (serum)	309	mOsm/kg	280–295
Blood urea nitrogen	12.4	mg/dL	8–20
Creatinine	0.7	mg/dL	0.6–1.2
Sodium	136	mEq/L	135–150
Potassium	3.6	mEq/L	3.5–5.5
Chloride	102	mEq/L	98–106
Albumin	5.2	g/dL	3.8–5.1
CK	453	IU/L	32–187
Blood ketone	8990	μmol/L	28–120
pH (ABGA)	6.92		7.36–7.41
PCO2 (ABGA)	12	mm Hg	35–48
PO2 (ABGA)	113	mm Hg	83–108
HCO3- (ABGA)	4.4	mmol/L	18–23
Anion gap	29.6	mEq/L	4–11
HbA1c	11.5	%	4.0–5.7
C-peptide	0.541	ng/mL	1.1–4.4

ABGA = arterial blood gas analysis, CK = creatine phosphokinase, HbA1c = glycated hemoglobin.

Based on these findings, the patient was diagnosed with metabolic acidosis due to DKA and immediate treatment was initiated. Aggressive hydration (>300 mL/h) and intravenous insulin therapy using an infusion pump were administered. The patient was transferred to the intensive care unit (ICU), remaining alert at the time of transfer. To prevent psychotic exacerbation during ICU care, clozapine was administered. Three hours after ICU admission, the patient’s mental status worsened to a stuporous state and respiratory failure developed, leading to oxygen desaturation and worsening acidosis. Immediate intubation was performed, and mechanical ventilation in synchronized intermittent mandatory ventilation mode was initiated. A non-contrast brain CT was performed to evaluate potential acute brain lesions; however, the findings were unremarkable. A fever of 39.3°C, which was absent at admission, developed and was treated with antipyretics and empirical antibiotics. On the second day of hospitalization, despite adequate hydration and insulin therapy stabilize blood glucose levels, the metabolic acidosis showed little improvement. Additionally, hyperthermia exceeding 39°C persisted despite antipyretic treatment. Diffusion-weighted brain magnetic resonance imaging was performed to evaluate the patient’s mental status; however, the results were unremarkable. No seizures or significant limb rigidity were observed. Blood pressure fluctuated markedly (systolic range: 100–160 mm Hg), and persistent tachycardia exceeding 130 beats/minute was noted.

**Figure 1.**
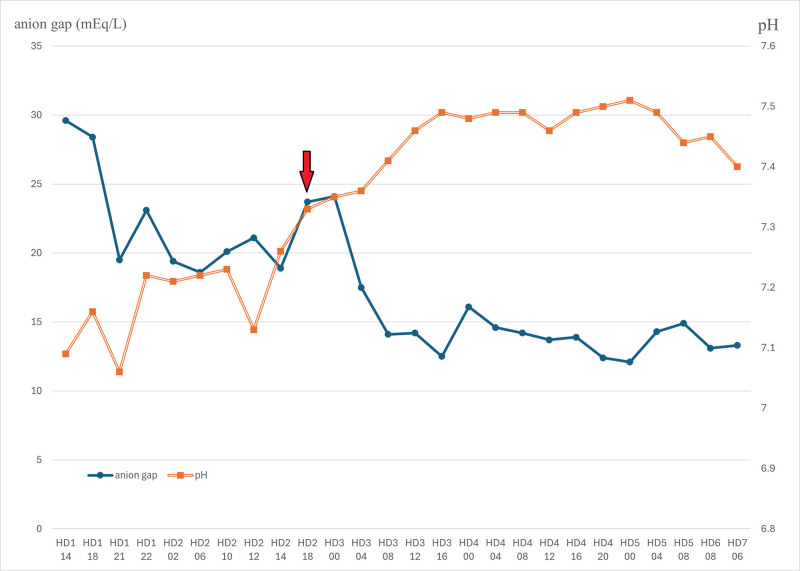
Changes in pH and anion gap during the hospitalization. X-axis, time marked with hospital day and time (24 h format). Arrow, clozapine discontinuation time.

Based on the patient’s medication history and recent switch to clozapine, close consultations were conducted with psychiatrists and neurologists. Given the persistent fever without a definite focus, stuporous mental status, respiratory failure, blood pressure fluctuations, and autonomic instability, NMS was suspected. Clozapine was discontinued and supportive care with insulin and hydration was continued. By the afternoon of the third hospital day, the fever had begun to decrease, and metabolic acidosis showed rapid improvement (Fig. 1). By the fourth hospital day, the patient’s mental status improved, and ventilator weaning was successfully initiated. On the fifth day of hospitalization, the endotracheal tube was removed. After extubation, mild aspiration was observed during fluid intake, necessitating Levin-tube feeding.

Multiple daily doses of insulin injections (MDI) were initiated on the sixth hospital day. On the eighth day of hospitalization, the patient was transferred to a general ward and adjustments for diabetes medications and insulin doses were made. Dysphagia gradually improved, and on the tenth hospital day, the Levine tube was removed, and oral feeding was resumed. Insulin requirements progressively decreased, allowing discontinuation of MDI on the seventeenth hospital day. On the nineteenth hospital day, the patient was discharged on basal insulin and oral diabetes medications, with plans for outpatient follow-up.

Meanwhile, at the time of admission, creatine kinase (CK) levels were elevated and continued to increase until the fourth hospital day despite adequate hydration therapy. However, the extent of CK elevation was relatively mild compared with the typically high levels observed in classic NMS cases. The initial C-peptide levels decreased but recovered by the tenth hospital day (Table 2).

**Table 2:** Changes in CK levels and C-peptide during treatment.

Date	CK	C-peptide
HD1	453	0.54
HD2	468	
HD3	544	
HD4	899	
HD5	652	
HD8	390	
HD11		2.06
HD16	131	

CK = creatine phosphokinase, HD = hospital day.

## 3. Discussion

Clozapine is used to treat treatment-resistant psychosis, particularly in patients that are partially or entirely unresponsive to other antipsychotic drugs. It is also effective in reducing violent and aggressive behavior in patients with schizophrenia.^[13,14]^ However, clozapine has pronounced effects on weight gain and abdominal obesity which carries a higher metabolic risk than other second-generation antipsychotics.^[6]^ In a 5-year observational study, clozapine-treated patients experienced the most significant weight gain between 12 and 46 months of treatment, with 36.6% developing diabetes.^[15]^ In particular, clozapine increased visceral fat, a hallmark of abdominal obesity, is strongly associated with insulin resistance.^[16]^

Clozapine’s actions on multiple receptors, including histamine H1 receptor (H1), serotonin 5-hydroxytryptamine 2A receptor (5-HT2A), serotonin 5-hydroxytryptamine 2C receptor (5-HT2C), dopamine D2 receptor (D2R), and melanocortin 4 receptor (MC4R), have been linked to its weight-gain effects. By modulating these receptors, clozapine reduces leptin activity, increases ghrelin levels, and enhances neuropeptide Y (NPY) expression, ultimately promoting appetite and reducing lipolysis, leading to visceral fat accumulation.^[17–20]^ Eventually, visceral fat accumulation can contribute to insulin resistance significantly.

Although weight gain and visceral fat contribute significantly to insulin resistance and diabetes, not all patients receiving clozapine experience weight gain. Therefore, weight gain alone cannot fully explain clozapine-induced glucose dysregulation and diabetes.^[21]^ Other hypotheses have been proposed concerning the mechanisms through which clozapine increases insulin resistance.

First, sphingolipids are components of the cell membrane. Ceramide is a bioactive sphingolipid that regulates cell cycle arrest, apoptosis, growth, survival, differentiation, aging, and stress responses.^[22]^ Ceramide is synthesized via 3 main pathways, namely, de novo, sphingomyelin, and salvage and recycling pathways. An excessive supply of saturated or unsaturated fatty acids can lead to ceramide accumulation, which reduces the activation of protein kinase B (PKB)/Akt, lowers glucose transporter type 4 expression, inhibits glucose uptake into cells, and disrupts insulin signaling, ultimately contributing to insulin resistance.^[23]^ In a mouse study, clozapine administration was associated with hyperglycemia and reduced glycogen levels, and a rapid decrease in hepatic ceramide content was observed within the first hour. Concurrently, ceramide synthase 2 protein levels and elongation of very long chain fatty acids protein 1 – enzymes involved in ceramide synthesis – increased. This led to the hypothesis that rapid ceramide depletion caused by clozapine disturbs sphingolipid homeostasis and may chronically enhance ceramide synthesis.^[24]^

Second, mitochondrial dysfunction is a known contributor to metabolic disorders. Oxidative stress from reactive oxygen species (ROS) and disruption of adenosine triphosphate (ATP) synthesis pathways can impair mitochondrial function.^[25]^ Clozapine has been shown to increase ROS production, thereby damaging mitochondria and creating a positive feedback loop that further promotes ROS generation.^[26]^ When ATP is depleted and the adenosine monophosphate (AMP)/ATP ratio increases, AMP-activated protein kinase (AMPK) is activated. Phosphorylated AMPK stimulates glucose uptake and fatty acid oxidation in the liver and muscle,^[27]^ whereas it suppresses glucose uptake and lipid metabolism in the hypothalamus. In experiments using mice and cell lines, clozapine inhibited AMPK activity in the liver while enhancing such activity in the brain.^[26]^ This finding supports the hypothesis that clozapine may induce metabolic dysfunction via mitochondrial impairment.

Third, increased levels of pro-inflammatory cytokines can lead to chronic inflammation, insulin resistance, and impaired glucose metabolism.^[28]^ In a mouse and human study, clozapine administration resulted in decreased glucagon levels and elevated expression of interleukin-1β (IL-1β), IL-6, and tumor necrosis factor-α (TNF-α).^[29]^ IL-1β binds to IL-1 receptors on pancreatic beta cells, potentially leading to cell apoptosis and impaired insulin secretion. It also binds to IL-1 receptors in the liver, contributing to hepatic steatosis and inhibition of hepatic insulin action.^[28]^ IL-6 and TNF-α induce insulin resistance by interfering with insulin receptor substrate-1, a key protein in the pathway activating PKB/Akt.^[30,31]^

While various hypotheses have been proposed to explain how clozapine induces insulin resistance, the exact mechanisms have yet to be determined. Therefore, further studies are needed to clarify the mechanisms involved.

Eventually, increased insulin resistance can induce DKA which is severe complications of diabetes.^[5]^ Clozapine-induced DKA typically occurs within an average of 5.8 weeks after treatment initiation. Studies have shown that patients who develop DKA tend to be younger and less overweight than those who develop only diabetes.^[32]^ In this case, the patient’s weight gain began a year earlier, but DKA developed within 2 months of switching to clozapine. Although weight gain may contribute to the development of diabetes, the timing strongly suggests that clozapine triggered DKA. Additionally, the patient’s c-peptide levels were low at presentation, but recovered after clozapine discontinuation, suggesting that clozapine may have contributed to impaired insulin secretion.^[33,34]^

On the other hand, the mechanisms by which antipsychotic drugs induce NMS are complex and are not entirely understood. Generally, NMS is considered to result from a sudden decrease in central dopaminergic activity due to D2 receptor blockade.^[35]^ Clozapine has a relatively low affinity for D2R but a higher affinity for dopamine D4 receptor (D4R), 5-HT2A, 5-HT2C, serotonin 5-hydroxytryptamine 6 receptor (5-HT6), serotonin 5-hydroxytryptamine 7 receptor (5-HT7), and H1 receptors. Additionally, clozapine acts as an antagonist of the D2R and 5-HT2A receptors in the nigrostriatal pathway and exhibits higher mesolimbic selectivity. These characteristics may explain the lower incidence of extrapyramidal side effects of clozapine and the atypical features of clozapine-induced NMS.

Various diagnostic criteria for NMS have been proposed to date. DSM-IV criteria (Table S1, Supplemental Digital Content, https://links.lww.com/MD/P782) stipulate that both fever and muscle rigidity are essential features, in addition to at least 2 of the following supportive features: diaphoresis, elevated or labile blood pressure, tachycardia, incontinence, dysphagia, mutism, tremor, altered level of consciousness ranging from confusion to coma, leukocytosis, and laboratory evidence of muscle injury (e.g., elevated CK).^[36]^ However, cases of atypical NMS that lack fever,^[37]^ muscle rigidity,^[36]^ or elevated CK have been reported.^[38–40]^ Nierenberg criteria (Table S2, Supplemental Digital Content, https://links.lww.com/MD/P782) define 5 major features – fever, muscle rigidity, elevated CK, autonomic instability, and altered mental status – and 4 minor features including other autonomic symptoms (e.g., incontinence, arrhythmia, diaphoresis), other extrapyramidal signs (e.g., tremor, cogwheeling, acute dystonic reaction, or choreiform movements), respiratory abnormalities (e.g., severe dyspnea, tachypnea, respiratory failure, or hypoxia), and leukocytosis. A diagnosis requires the presence of either all 4 major criteria or 3 major criteria plus 3 or more minor criteria.^[41]^ Adityanjee et al’s diagnostic criteria for NMS (Table S3, Supplemental Digital Content, https://links.lww.com/MD/P782) account for the possibility of atypical NMS presentations associated with second-generation antipsychotics, which may lack or present minimal muscle rigidity.^[42]^ The DSM-5 criteria (Table S4, Supplemental Digital Content, https://links.lww.com/MD/P782) include similar diagnostic elements to the DSM-IV but do not specify the number of required criteria components,^[36]^ thus allowing for a broader clinical diagnostic assessment.

The patient had received clozapine within the preceding 72 hours and exhibited persistent fever, altered mental status not attributable to other causes, autonomic instability (tachycardia and blood pressure fluctuations), leukocytosis, and respiratory failure. A diagnosis of NMS was made based on DSM-5, Nierenberg, and Adityanjee et al criteria.

Typical NMS symptoms include rigidity, hyperthermia, and elevated CK levels. However, atypical NMS caused by clozapine is characterized by the absence of rigidity and delayed CK elevation in several reported cases.^[38–40,43]^ In this patient, hyperthermia, altered consciousness, and autonomic instability were observed; however, severe rigidity and sharp increase in CK levels were absent. This presentation aligns with the previously reported atypical features of clozapine-induced NMS.^[44]^

Reports on the simultaneous occurrence of DKA and NMS in patients treated with antipsychotic drugs are extremely rare. A case involving a patient treated with chlorpromazine and zuclopenthixol was reported in 1992,^[45]^ and another involving a patient treated with olanzapine in 2013.^[12]^ While clozapine-induced DKA or NMS has been reported separately in many cases, the concurrence of both conditions has not been documented so far. Compared to a previous case involving olanzapine,^[12]^ where hyperthermia, rigidity, and sharply elevated CK levels were observed as typical features of NMS, our case showed the absence of typical features.

Hallmark symptoms of NMS, such as hyperthermia, altered consciousness, blood pressure instability, dyspnea, and tachycardia, are commonly observed in DKA.^[10,11]^ As a result, in cases in which one of the conditions is confirmed, the possibility of the other may be easily overlooked. When the clinical history, physical examination, and laboratory findings raise suspicion, a differential diagnosis is essential. While there is no clear evidence that DKA and NMS influence each other’s onset, severe stress conditions such as infection, trauma, or cardiovascular emergencies are known risk factors for DKA.^[46]^ On the other hand, iron deficiency, trauma, and infections have been suggested as risk factors for NMS, the relationship between NMS and acute medical stress has not been universally substantiated.^[47–50]^ Although these 2 conditions develop independently, they can negatively affect the course of each other. Therefore, rapid differentiation and appropriate management of both conditions are crucial.^[51,52]^

The patient had been taking ropinirole, benztropine, quetiapine, and amisulpride along with clozapine up until the day prior to admission; therefore, the influence of other psychiatric medications cannot be entirely excluded. However, these medication doses were either reduced or maintained when clozapine was introduced. Moreover, as clozapine was the last medication administered prior to the onset of symptoms, we deemed it likely that clozapine played a primary role in the development of NMS. NMS has previously been reported to occur in patients treated with both clozapine and quetiapine,^[8]^ as well as during cross-titration from quetiapine to clozapine.^[53]^ Therefore, caution is warranted when clozapine is used concurrently with or substituted for other antipsychotics.

In this case, despite being in a state of severe metabolic acidosis, the patient maintained an alert mental status and relatively stable respiratory function upon admission. However, within a few hours, the patient’s condition rapidly deteriorated to a stuporous mental status and respiratory failure despite intensive hydration and insulin therapy for DKA. Moreover, hyperthermia, metabolic acidosis, and altered mental status persisted, even after blood glucose levels were stabilized. Once the possibility of NMS was considered and clozapine was discontinued, the patient’s fever, metabolic acidosis, and mental status improved significantly, allowing for ventilator weaning. This case also showed that delayed recovery from DKA due to concomitant NMS can induce life-threatening respiratory failure.

As demonstrated in this case, patients on clozapine are at a higher risk of metabolic syndrome and its associated complications, such as obesity, hypertension, hyperglycemia, and dyslipidemia, making regular monitoring essential. In Victoria, Australia, a consensus recommends monitoring BMI and waist circumference every 3 months, blood glucose every 3 to 6 months, and blood pressure and lipid levels every 6 months for patients on clozapine who are at risk of diabetes.^[54]^ American and European guidelines suggest measuring BMI every 4 weeks during the initial 12 weeks of clozapine therapy, along with lipid testing at weeks 4 and 12.^[55–59]^ Similarly, the U.S. food and drug administration advises routine assessments of glucose levels, body weight, and symptoms of hyperglycemia in patients receiving clozapine treatment.^[60]^

## 4. Conclusion

We describe a patient who developed simultaneous DKA and atypical NMS while on clozapine therapy, accompanied by delayed recovery from ketoacidosis. The patient’s condition improved with discontinuation of clozapine, hydration, insulin therapy, and supportive care. This case highlights the importance of considering the possibility of NMS in patients with DKA as well as evaluating metabolic syndrome and DKA in patients with NMS. Additionally, clozapine-induced NMS may present in atypical forms with minimal rigidity and CK elevation, emphasizing the need for careful review of the patient’s history and medication use for a differential diagnosis.

## Author contributions

**Investigation:** Min Hyeok Shin, Jin Woo Jeong.

**Methodology:** Jin Woo Jeong, Tae Yang Yu.

**Writing – original draft:** Min Hyeok Shin.

**Writing – review & editing:** Jin Woo Jeong, Tae Yang Yu.

## Supplementary Material


