# Supplementary material for: Concurrence of clozapine-induced diabetic ketoacidosis and neuroleptic malignant syndrome: A case report
Source: Medicine (Baltimore). 2025 Aug 22;104(34):e44172. doi: 10.1097/MD.0000000000044172 (PMC12385089; doi:10.1097/MD.0000000000044172)
Supplement: Supplementary file 1 [file medi-104-e44172-s001.pdf]

Supplemental Table S1. DSM-IV NMS criteria

Major criteria<br>-The development of severe muscle rigidity<br>-Elevated temperature associated with the use of antipsychotic medication
Minor criteria<br>-Diaphoresis<br>-Elevated or labile blood pressure<br>-Tachycardia<br>-Incontinence<br>-Dysphagia<br>-Mutism<br>-Tremor<br>-Changes in the level of consciousness ranging from confusion to coma<br>-Leukocytosis<br>-Laboratory evidence of muscle injury (e.g., elevated CK)
-The symptoms are not due to another substance, neurologic or other medical conditions<br>-The symptoms are not better accounted for by a mental disorder
Number of required criteria<br>Both two items of major criteria and at least two items of minor criteria

DSM-IV: Diagnostic and Statistical Manual of Mental Disorders, Fourth Edition, NMS: neuroleptic malignant syndrome, CK: creatinine phosphokinase, EPS: extrapyramidal side effects

Supplemental Table S2. NMS diagnostic criteria by Nierenberg et al., 1991

Essential criteria<br>-Receiving or recently received a neuroleptic drug<br>-Receiving other dopamine antagonist<br>-Recently stopped therapy with a dopamine agonist
Major criteria<br>-Hyperthermia (temperature 38" C or higher without other obvious source)<br>-Muscular lead-pipe rigidity<br>-Altered consciousness (e.g., obtundation, stupor, coma, delirium, or mutism)<br>-Marked autonomic dysregulation (two or more: sweating, PR > 100/min, BP > 150/100 or BP < 90/60 mm Hg)<br>-Elevated CK (>3 times normal without other obvious cause)
Minor criteria<br>-Other signs of EPS (e.g., tremor, cogwheeling, acute dystonic reaction, or choreiform movements)<br>-Other manifestations of autonomic dysfunction (e.g., urinary incontinence, arrhythmias, or one of sweating, P > 100, BP > 150/100 or BP < 90/60 mm Hg not counted above)<br>-Respiratory problems (e.g., severe dyspnea, tachypnea, respiratory failure, or hypoxemia)<br>-Leukocytosis (WBC count >12.0 x 10^9 per liter)
Diagnosis needs 1 essential criteria and 4 major criteria or 3 major criteria plus three minor criteria.

NMS: neuroleptic malignant syndrome, CK: creatinine phosphokinase, EPS: extrapyramidal side effects

Supplemental Table S3. Diagnostic criteria for NMS by Adityanjee et al., 1999

(1) Altered sensorium (any one of the following)<br>-Confusion<br>-Clouding of consciousness<br>-Mutism<br>-Stupor<br>-Coma	(3) Hyperpyrexia of unknown origin<br>-Should be greater than 38.5 °C<br>-Should be sustained for at least 48 h in duration<br>-No concurrent physical/medical cause for hyperpyrexia
(2) Extrapyramidal motor symptoms (any one of the following)<br>-Muscular rigidity<br>-Dysphagia<br>-Dystonia	(4) Autonomic dysfunction (at least 2 of the following)<br>-Tachycardia (pulse more than 100/min)<br>-Tachypnoea (respiration more than 25/min)<br>-Blood pressure fluctuations (at least a change of 30 mmHg in systolic pressure or 15 mmHg in diastolic pressure)<br>-Excessive sweating (diaphoresis)<br>-New onset incontinence
(5) Relationship of onset of symptoms with exposure event defined by any one of the following<br>-Oral ingestion or parenteral administration (dose increase, dose decrease, discontinuation) of an antipsychotic drug (typical or atypical), a dopamine depleter dopamine blocker or a psychostimulant drug during the previous 2 weeks<br>-Withdrawal of antiparkinsonian or anticholinergic drug during previous 1 week<br>-Intramuscular administration of a long-acting depot antipsychotic medication during the previous 8 weeks	
(6) Exclusion criteria Symptoms not due to any other existing or new general medical (secondary to substance abuse, infectious illnesses, metabolic, delirium, etc.), neurologic (encephalitis, epilepsy, brain tumours, etc.) or psychiatric disorder (e.g. catatonic schizophrenia, mood disorder with catatonic features).	(7) Supportive features (any two of the following)<br>-Elevations in serum CK levels<br>-Leukocytosis<br>-Low serum iron levels<br>-Elevation of liver enzymes<br>-Myoglobinuria
Typical (Classical) NMS: Criteria (1)-(6) must be present<br>Atypical NMS: Criteria numbers (1), (3) and (4), (5), (6) and any one item from criteria number (7) must be present for the diagnosis. Criteria number (2) is not necessary for making diagnosis.	

NMS: neuroleptic malignant syndrome, CK: creatinine phosphokinase, EPS: extrapyramidal side effects

Supplemental Table S4. DSM-V NMS criteria

Major symptoms<br>-Rigidity<br>-Hyperthermia<br>-Diaphoresis<br>-Exposure to dopamine antagonist within 72hour prior to the beginning of symptoms
Minor symptoms<br>-Autonomic nervous system instability<br>-Altered consciousness<br>-Tremor, akinesia, dystonia, myoclonia, trismus, dysarthria, dysphagia<br>-Leukocytosis<br>-CK, myoglobin, catecholamine, creatinine elevation<br>-Metabolic acidosis<br>-Hypoxia
Exclusion criteria<br>The above symptoms are not due to another substance or a neurological or other general medical condition.

DSM-5: Diagnostic and Statistical Manual of Mental Disorders, Fifth Edition, NMS: neuroleptic malignant syndrome, CK: creatinine phosphokinase, EPS: extrapyramidal side effects
